# LONELINESS AND PSYCHOLOGICAL WELL-BEING OF THE INSTITUTIONAL OLDER ADULTS DURING THE COVID-19 PANDEMIC

**DOI:** 10.1093/geroni/igac059.2631

**Published:** 2022-12-20

**Authors:** Shiau-Fang Chao, Hui-chuan Hsu, Yun-Pei Su

**Affiliations:** National Taiwan University, Taipei, Taipei, Taiwan (Republic of China); Taipei Medical University, Taipei, Taipei, Taiwan (Republic of China); Taipei Medical University, Taipei, Taipei, Taiwan (Republic of China)

## Abstract

**Purpose:**

Institutional residents experienced more restrictions in the lockdown of covid-19. The purpose of this study was to examine the association of loneliness and lonely literacy with mental health wellbeing during covid-19 for older institutional residents.

**Methods:**

The participants living in the 13 long-term care institutions who were aged 65 and more and able to communicate with were invited in the survey (n=143). Mental well-being was measured by depressive symptoms and life satisfaction. Loneliness was measured by the 6-item UCLA loneliness scale. In addition, demographics, health status, active and passive coping strategies, social support from family and friends, social interaction changes after covid-19, loneliness change after covid-19, and worries about covid-19 were investigated. Linear regression and logistic regression models were conducted.

**Results:**

The mean of the loneliness score (6~24) was 9.71 (SD=4.02). Factors related to loneliness increased during covid-19 for the institutionalized residents included having more physical function difficulties (OR=1.179), feeling more lonely (OR=1.146), and having more worries for covid-19 (OR=2.317). The residents having depressive symptoms was related to have more loneliness (OR=1.269), worse self-rate health (OR=0.320), and increased more loneliness during covid-19 (OR=3.233); while having high life satisfaction was related to less loneliness (OR=0.859), less physical difficulties (OR=0.834), higher satisfaction of family support (OR=2.835), and not increasing loneliness during covid-19 (OR=0.255).

**Conclusion:**

Loneliness during covid-19 is related to more depressive symptoms and lower life satisfaction, especially during covid-19. Learning active coping strategy and providing proactive and helpful environment for the long-term care residents is suggested during the pandemics.

